# Transcatheter Aortic Valve Repair in a Patient With Anomalous Right Coronary Artery Originating From the Left Aortic Sinus and Myelodysplastic Syndrome

**DOI:** 10.7759/cureus.9073

**Published:** 2020-07-08

**Authors:** Katharina Birkl, Fabian Plank, Nikos Bonaros, Gudrun Feuchtner, Guy Friedrich

**Affiliations:** 1 Radiology, Medical University of Innsbruck, Innsbruck, AUT; 2 Cardiology, Medical University of Innsbruck, Innsbruck, AUT; 3 Cardiac Surgery, Medical University of Innsbruck, Innsbruck, AUT

**Keywords:** transcatheter aortic valve repair, cardiac computed tomography, coronary artery bypass graft surgery

## Abstract

We report a patient with symptomatic low-flow high-grade aortic valve stenosis and myelodysplastic syndrome. Preinterventional imaging revealed an anomalous origin of the right coronary artery only defined by CT. The patient was classified as high risk in regard to conventional cardiac surgery by our heart team and therefore scheduled for transcatheter aortic valve repair (TAVR). The case presentation describes the potential effect of this anatomical coronary variant with regard to the peri- and postinterventional outcome: anomalous origin of the right coronary artery may lead to severe ischemia during TAVR due to occlusion of the coronary vessel. Conversion to open surgery with immediate coronary bypass surgery may rapidly restore myocardial perfusion and enhance clinical outcome of the patient.

## Introduction

Transcatheter aortic valve repair (TAVR) has become the preferred therapy in patients with symptomatic high-grade aortic valve (AV) stenosis and for whom open heart surgery is contraindicated. Excellent peri- and postoperative outcomes for TAVR patients at high or intermediate surgical risk have led to a more frequent use of this therapy at many centers [1-2]. Nevertheless, unique anatomical features may have a significant impact on the clinical outcome in TAVR patients. Therefore, careful pre- and perioperative monitoring is required to attain optimal interventional results. In selected cases, conversion to open heart surgical repair may be required in unpredictable emergencies. We report a patient with symptomatic low-flow high-grade AV stenosis and myelodysplastic syndrome (MDS). The patient was scheduled for a TAVR procedure, and preinterventional imaging revealed an anomalous origin of the right coronary artery (RCA). We describe the potential effect of this anatomical coronary variant concerning the peri-and postinterventional outcome.

## Case presentation

**Figure 1:**
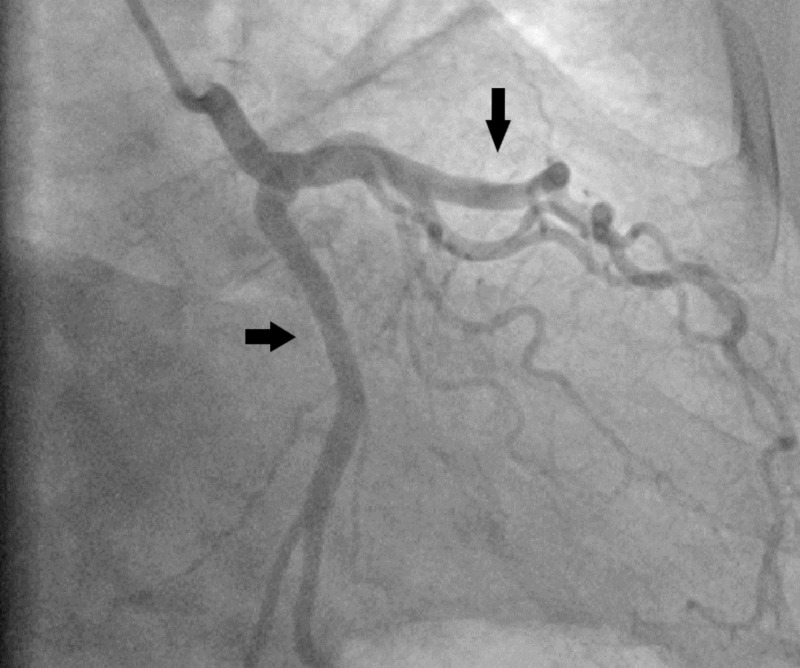
Selective angiography of left coronaries (arrows showing non-obstructive left anterior descending and circumflex arteries)

**Figure 2:**
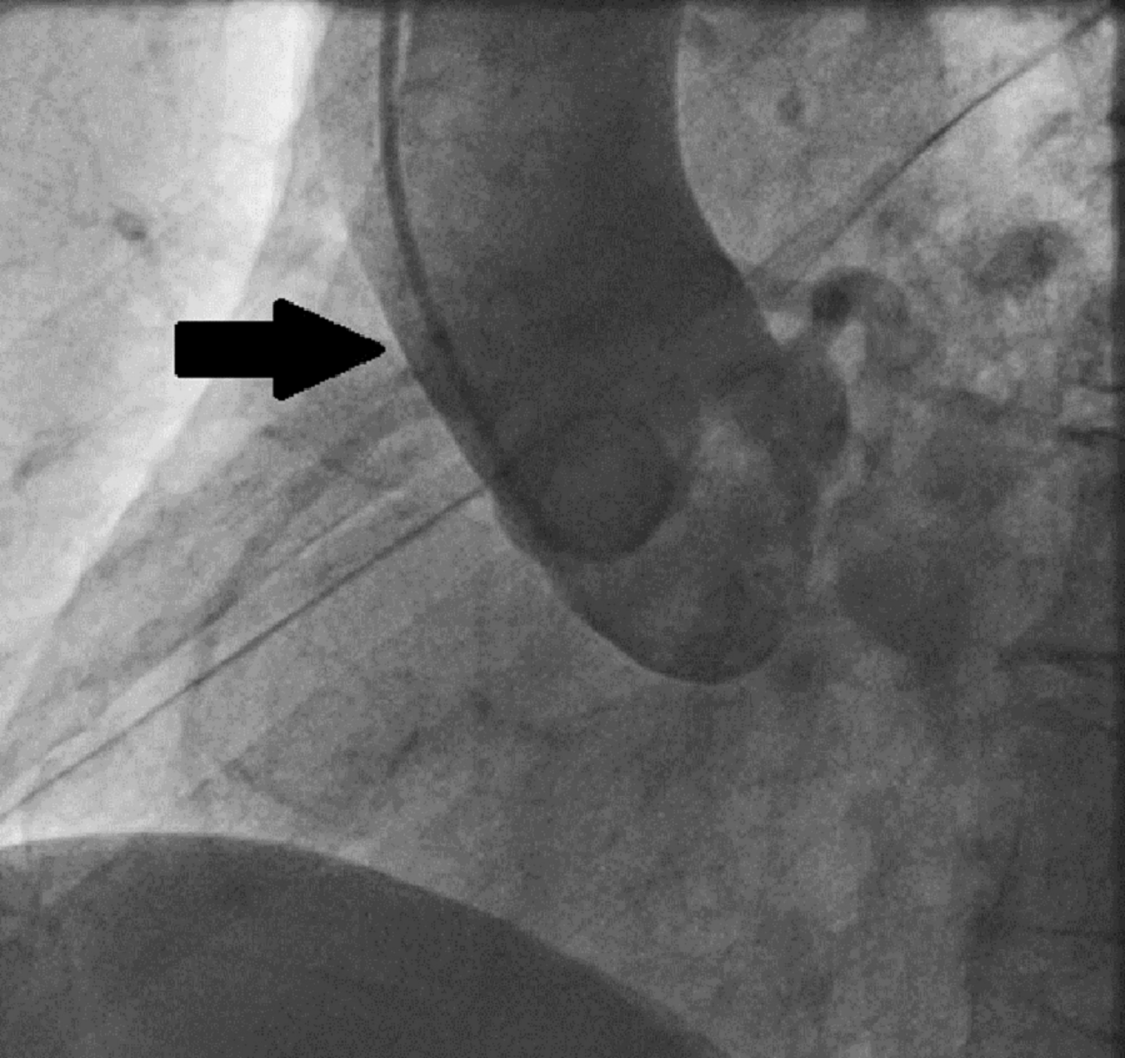
Indirect angiographic visualization of the right coronary artery by supra-aortal pigtail catheter contrast injection (arrow showing anomalous origin of the right coronary artery)

**Figure 3:**
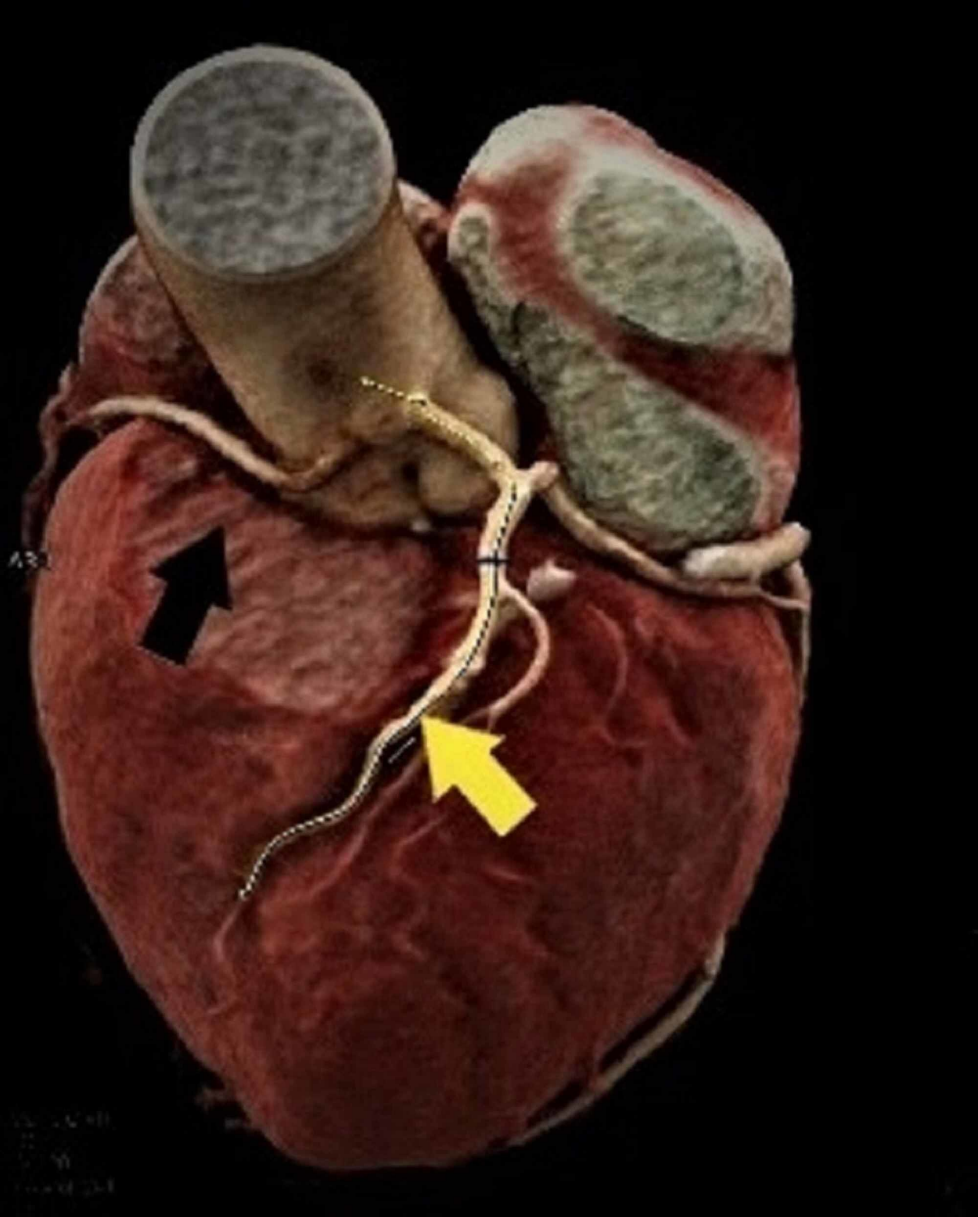
Three-dimensional CT imaging of the origin and course of the right coronary artery (black arrow) and the left coronary artery (yellow arrow)

A 76-year-old man presented to our cardiology department with atypical chest pain and shortness of breath. Physical examination revealed a loud 4/6 systolic murmur related to the AV. Transthoracic echocardiography showed low-flow high-grade AV stenosis (mean AV gradient of 40 mm Hg, aortic orifice area of 0.75 cm2). Former clinical history included MDS that was first diagnosed in 2018 with leukopenia, thrombopenia, and anemia but no actual need for chemotherapy or transfusion of blood cells or plasma. Invasive coronary angiography and multislice CT following a specific TAVR protocol, as well as a transesophageal echocardiography (TEE), were performed. Coronary angiography revealed diffuse coronary sclerosis without significant stenoses in the left anterior descending artery and circumflex vessels. Despite the use of different diagnostic coronary catheter types and selective intubation, visualization of the RCA could not be achieved due to a suspected anomalous origin of the vessel (Figures 1, 2). Pre-TAVR CT angiography confirmed an anomalous stenotic origin of the RCA from the left aortic sinus (Figure 3).

 

Due to the comorbidities related to MDS and the high probability of infectiologic complications occurring after conventional open valve surgery, the heart team at our institution decided to perform a transfemoral TAVR procedure. The intervention was achieved using a balloon-expandable 26-mm Edwards Sapien 3 transcatheter valve (Edwards Lifesciences, Irvine, CA, USA). The choice of the bioprosthetic valve type was based on local expertise and suitable anatomical features such as degree and extent of valve calcification.

After the release of the transcatheter valve, unspecific electrocardiogram (ECG) ST-segment depression alterations occurred with hemodynamic worsening and a decrease in systolic blood pressure to 70 mm Hg. Severe right ventricular dysfunction with unchanged left ventricular ejection fraction (EF) was revealed on TEE. The right ventricular wall motion was reduced, and right ventricle dilatation was recorded. There were no signs of pericardial effusion or peripheral bleeding. The TAVR valve was judged to be in the correct position, and no AV regurgitation was present.

**Figure 4:**
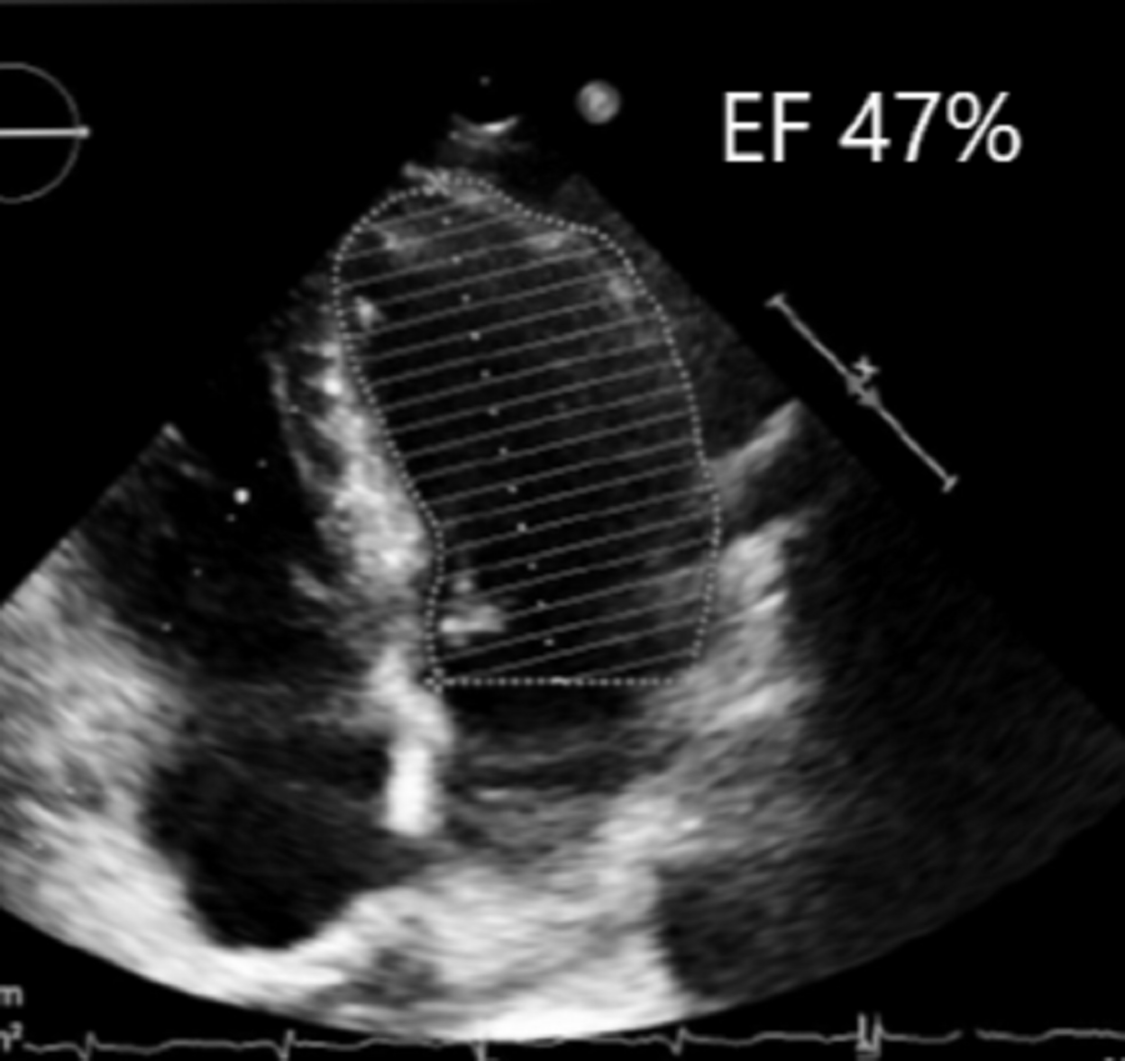
Two-dimensional transthoracal echocardiography showing left ventricular ejection fraction of 47%

Fluid substitution and high-dosage catecholamine infusions were administered, although no clinical benefit was observed. Due to persistent ECG ST-segment changes, we suspected an ischemic right ventricular infarction and occlusion of the RCA. Following an acute worsening of hemodynamics, we opted for intervention consisting of a conversion to sternotomy and a single venous bypass graft surgery of the RCA. The patient’s hemodynamic situation rapidly stabilized, and postoperative weaning was unproblematic. A subsequent echocardiography revealed minimal reduction of global left ventricular EF (47%), minimal AV regurgitation, and no remaining AV stenosis (Figure 4).

## Discussion

An anomalous coronary artery originating from the opposite sinus (ACAOS) of Valsalva may lead to hemodynamic and ischemic complications, especially in TAVR patients. The CT diagnosis and incidence of ACAOS anatomy have been previously described and are related to potential clinical outcomes. There is poor evidence considering the influence of ACAOS anatomy in TAVR procedures [3-6]. In this case, a more malignant variable with pulsatile compression between the aortic root and left ventricular outflow may have led to a complete occlusion of the RCA after TAVR. This complication could have been fostered by a pre-existing sclerotic or pulsatile-mediated functional stenosis of the vessel. Coronary obstruction is a reported rare but severe complication in TAVR patients. Despite the fact that percutaneous coronary revascularization is feasible in many cases, the mortality rate in these patients is high. Pre-TAVR imaging therefore is mandatory to avoid peri- interventional myocardial ischemia [7-9]. Percutaneous coronary stenting of the RCA in ACAOS patients, however, is not a satisfactory option because a reasonable guiding catheter position is anatomically impossible. Therefore, coronary bypass combined with AV open heart surgery may be the preferred strategy. Our patient’s comorbidities related to MDS were considered as a contraindication for this approach.

## Conclusions

In patients with anomalous RCA origin, TAVR may lead to myocardial ischemia with poor interventional therapeutic options. Careful consideration of TAVR should be mandatory for these patients, especially in the presence of dominant RCA anatomy. When patients develop severe hemodynamic complications during the TAVR procedure, conversion to emergency bypass surgery may be the only therapeutic alternative.
